# Pharmacologic Inhibition of Myostatin With a Myostatin Antibody Improves the Skeletal Muscle and Bone Phenotype of Male Insulin‐Deficient Diabetic Mice

**DOI:** 10.1002/jbm4.10833

**Published:** 2023-10-26

**Authors:** R Clay Bunn, Reuben Adatorwovor, Rebecca R Smith, Philip D Ray, Sarah E Fields, Alexander R Keeble, Christopher S Fry, Sasidhar Uppuganti, Jeffry S Nyman, John L Fowlkes, Evangelia Kalaitzoglou

**Affiliations:** ^1^ Department of Pediatrics and Barnstable Brown Diabetes Center University of Kentucky Lexington KY USA; ^2^ Department of Biostatistics, College of Public Health University of Kentucky Lexington KY USA; ^3^ Sanders‐Brown Center on Aging University of Kentucky Lexington KY USA; ^4^ Department of Pediatrics University of Kentucky Lexington KY USA; ^5^ College of Agriculture, Food and Environment University of Kentucky Lexington KY USA; ^6^ Center for Muscle Biology University of Kentucky Lexington KY USA; ^7^ Department of Orthopaedic Surgery Vanderbilt University Medical Center Nashville TN USA; ^8^ Department of Veterans Affairs Tennessee Valley Healthcare System Nashville TN USA

**Keywords:** BONE, MUSCLE, MYOSTATIN, TYPE 1 DIABETES MELLITUS

## Abstract

Type 1 diabetes (T1D) is associated with low bone and muscle mass, increased fracture risk, and impaired skeletal muscle function. Myostatin, a myokine that is systemically elevated in humans with T1D, negatively regulates muscle mass and bone formation. We investigated whether pharmacologic myostatin inhibition in a mouse model of insulin‐deficient, streptozotocin (STZ)‐induced diabetes is protective for bone and skeletal muscle. DBA/2J male mice were injected with low‐dose STZ (diabetic) or vehicle (non‐diabetic). Subsequently, insulin or palmitate Linbits were implanted and myostatin (REGN647‐MyoAb) or control (REGN1945‐ConAb) antibody was administered for 8 weeks. Body composition and contractile muscle function were assessed in vivo. Systemic myostatin, P1NP, CTX‐I, and glycated hemoglobin (HbA1c) were quantified, and gastrocnemii were weighed and analyzed for muscle fiber composition and gene expression of selected genes. Cortical and trabecular parameters were analyzed (micro‐computed tomography evaluations of femur) and cortical bone strength was assessed (three‐point bending test of femur diaphysis). In diabetic mice, the combination of insulin/MyoAb treatment resulted in significantly higher lean mass and gastrocnemius weight compared with MyoAb or insulin treatment alone. Similarly, higher raw torque was observed in skeletal muscle of insulin/MyoAb‐treated diabetic mice compared with MyoAb or insulin treatment. Additionally, muscle fiber cross‐sectional area (CSA) was lower with diabetes and the combination treatment with insulin/MyoAb significantly improved CSA in type II fibers. Insulin, MyoAb, or insulin/MyoAb treatment improved several parameters of trabecular architecture (eg, bone volume fraction [BV/TV], trabecular connectivity density [Conn.D]) and cortical structure (eg, cortical bone area [Ct. Ar.], minimum moment of inertia [Imin]) in diabetic mice. Lastly, cortical bone biomechanical properties (stiffness and yield force) were also improved with insulin or MyoAb treatment. In conclusion, pharmacologic myostatin inhibition is beneficial for muscle mass, muscle function, and bone properties in this mouse model of T1D and its effects are both independent and additive to the positive effects of insulin. © 2023 The Authors. *JBMR Plus* published by Wiley Periodicals LLC on behalf of American Society for Bone and Mineral Research.

## Graphical abstract

This study on male insulin‐deficient diabetic mice treated with insulin or a myostatin inhibitory antibody demonstrates that myostatin inhibition is beneficial for muscle and bone and that it can have additive effects to insulin therapy. These data indicate that myostatin is a potential target for diabetic bone disease and diabetic myopathy.

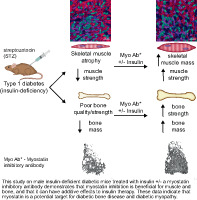

## Introduction

The prevalence of type 1 diabetes (T1D) in those younger than 20 years of age has risen significantly;^(^ ^1^ ^)^ therefore, prevention and therapy of diabetic complications is of high importance to improve clinical outcomes and complication‐associated costs.

Diabetic bone disease (DBD), considered now a serious complication associated with T1D, is characterized by decreased bone mineral density,^(^ ^2^ ^)^ impaired bone microarchitecture,^(^ ^3^ ^)^ and an increase in risk of fracture.^(^ ^4^, ^5^ ^)^ Bone mass and bone size are negatively affected in adolescents with T1D,^(^ ^6^, ^7^ ^)^ and those with T1D exhibit fractures throughout the life span,^(^ ^8^ ^)^ despite advancements in insulin therapy. Furthermore, certain factors such as age, longer diabetes duration, and T1D diagnosis before peak bone mass accrual are risk factors for fractures in those with T1D.^(^ ^9^ ^)^ Lastly, although there is an increased risk of fracture associated with T1D, there are no clinical trials to evaluate antifracture therapy in patients with T1D, primarily due to the lack of information about underlying mechanisms and potential therapeutic targets. In addition to DBD, T1D is associated with deficits in skeletal muscle, also termed diabetic myopathy, a finding that is evident early on in the disease.^(^ ^10^, ^11^ ^)^ Specifically, atrophy of muscle fibers,^(^ ^12^ ^)^ reduced muscle strength and work performance,^(^ ^13^, ^14^ ^)^ and alterations in mitochondrial function^(^ ^15^ ^)^ have been reported in those with T1D.

Muscle mass and bone mass are closely related during development and growth, and muscle and bone interact through mechanical, endocrine, and paracrine factors in physiologic and disease states.^(^ ^16^, ^17^ ^)^ Myokines, which are factors secreted by skeletal muscle, contribute to muscle‐bone communication and have even been associated with direct effects on bone and bone cells.^(^ ^18^ ^)^ Myokines can be dysregulated in several disease states, including diabetes.^(^ ^19^ ^)^ Myostatin, which has been found to be systemically elevated in humans with T1D^(^ ^20^, ^21^ ^)^ is a member of the TGF‐β family. It is primarily secreted by skeletal muscle and plays a central role in muscle homeostasis, as it negatively regulates muscle mass.^(^ ^22^ ^)^ Animal models of insulin‐deficient diabetes show upregulation of myostatin,^(^ ^23^, ^24^, ^25^ ^)^ and inhibition of myostatin signaling in one of these studies has shown promising results in restoring the impaired regenerative responses in muscle associated with insulin‐deficient diabetes.^(^ ^25^ ^)^ Additionally, exercise‐induced downregulation of myostatin has been shown to be associated with activation of the Wnt/GSK‐3β/β‐catenin pathway in bone of diabetic rats,^(^ ^26^ ^)^ supporting a direct effect of myostatin on bone signaling in the diabetic state. The effects of pharmacologic myostatin inhibition on muscle and bone phenotype of insulin‐deficient diabetic mice have not been examined and information about myostatin's direct action on bone of diabetic mice is unclear.

In the present study, we examined the effects of pharmacologic inhibition of myostatin with a myostatin‐blocking antibody (REGN647^(^ ^27^, ^28^ ^)^) in the skeletal muscle and bone properties of an animal model of insulin‐deficient (T1D) diabetes, with and without insulin treatment. Additionally, we examined whether myostatin has direct effects on mineralization and differentiation of murine pre‐osteoblasts (MC3T3‐E1).

## Materials and Methods

### Mouse study design

*Induction of diabetes and treatment arms*: Ten‐week‐old male DBA/2J mice (The Jackson Laboratory, Bar Harbor, ME, USA) received ip injections of streptozotocin (Sigma‐Aldrich, Burlington, MA, USA) at 40 mg/kg/d in citrate buffer (diabetic‐D) or citrate buffer alone (non‐diabetic‐ND). After confirming persistent hyperglycemia (non‐fasting blood glucose above 250 mg/dL), diabetic (D) mice were randomized to receive sustained‐release LinBit insulin implants (Ins) (LinShin Canada Inc, Scarborough, Canada) or blank palmitic acid micro‐crystal implants as control (Palm) (LinShin Canada Inc) under anesthesia. Non‐diabetic (ND) mice had control (Palm) LinBit implants inserted. All implants were inserted and replaced based on manufacturer's recommendations (http://www.linshincanada.com/linbit.html). LinBits were replaced if blood glucose >300 mg/dL over a period of 1 week in diabetic mice. Non‐diabetic mice had their control LinBits replaced once during the study. D and ND mice were further randomized to receive anti‐myostatin (REGN647‐MyoAb, Regeneron, Tarrytown, NY, USA) or Isotype control (REGN1945‐ConAb, Regeneron) antibody at 10 mg/kg once weekly for the first 4 weeks and then twice weekly for the remainder of the study. Both antibodies were given subcutaneously after brief anesthesia with isoflurane. Mouse weight was measured weekly and before euthanasia. Gastrocnemius weight was measured after euthanasia. All mice were maintained in a 14‐hour light/10‐hour dark cycle, and provided *ad libitum* access to chow diet (2018 Teklad, Envigo, Indianapolis, IN, USA) and water throughout the study. All animal procedures were approved by the University of Kentucky Institutional Animal Care and Use Committee.

### Serum assays

Whole blood was collected during euthanasia and was stored at −20°C or processed for serum isolation. Serum specimens were also stored at −20°C until ready to be assayed. Myostatin was measured in serum with a GDF‐8/Myostatin Quantikine ELISA kit (Cat# DGDF80, R&D Systems/Bio‐Techne, Minneapolis, MN, USA). P1NP was measured with Rat/mouse P1NP EIA assay kit (IDS, Boldon, UK, Cat# AC‐33F1), and RatLaps (CTX‐I) was measured with Rat/mouse EIA assay kit (IDS Cat# AC‐06F1). Glycated hemoglobin was measured in whole blood with an enzymatic mouse Hemoglobin A1c assay kit (Crystal Chem, Elk Grove Village, IL, USA, Cat# 80310).

### Body composition analysis

Mice underwent Echo‐MRI (EchoMRI‐100 [EMR‐102 2016]) scans to assess body composition parameters, including total body fat, lean mass, and total body water at the beginning of the study and before euthanasia. Conscious mice were individually restrained in a clear cylindrical plastic holder (sized by animal weight). The holders have holes for breathing and are maintained in the horizontal plane during the procedure. Each scan lasted approximately 2 minutes.

### In vivo plantar flexor peak torque

Before euthanasia, muscle function was assessed in a subgroup (*n* = 6/group) of diabetic mice. The strength of the plantar flexor muscle complex was assessed by in vivo isometric peak tetanic torque, similar to our prior published methods.^(^ ^29^ ^)^ In an induction chamber, mice were anesthetized with 2.5% isoflurane vaporized in 1.5 L/min oxygen (VetEquip [Livermore, CA, USA] vaporizer). Mice were then transferred to a secure nose cone with a continuous flow of isoflurane in oxygen. The right hindlimb was analyzed for all mice, and fur was trimmed (Wahl Bravmini, Wahl Corporation, Sterling, IL, USA) to ensure unobstructed electrode placement. Mice were placed in the supine position on a 37°C temperature‐regulated platform (809c in situ mouse apparatus, Aurora Scientific, Aurora, Canada), and the hindlimb was secured using a clamp at the knee with the foot placed in a footplate on a dual‐mode lever and motor (300D‐300C‐LRFP, Aurora Scientific). Surgical tape was wrapped around the foot secured to the footplate to prevent movement of the heel of placement shifting, and the footplate and motor arm was adjusted to place the tibia parallel with the platform with a 90‐degree angle at the ankle. Needle electrodes were positioned percutaneously slightly lateral to the knee to maximally stimulate the tibial nerve using an electrical stimulator (High Power Bi‐Phase Stimulator, Aurora Scientific). Using repeated twitches with the Instant Stimulation function with Live View in Dynamic Muscle Control LabBook (DMC v6.000), placement of needle electrodes was adjusted to optimize location to generate maximum isometric torque and eliminate antagonistic dorsiflexion. Optimal amperage to produce maximal torque was determined by a progressive series of twitch experiments (0.05‐second stimulus duration) beginning with 10 mA and increasing in small increments until the maxim torque stimulated by the minimum amperage was recorded. The amperage then remained constant throughout the force‐frequency experiment (10, 40, 80, 120, 150, 180, and 200 Hz, 0.25‐second stimulus duration with a 2‐minute rest period between each stimulus) from which isometric peak tetanic torque was recorded. Peak torque data were collected using DMC v6.000 and analyzed with Dynamic Muscle Analysis software (DMA v5.501). Plantar flexor isometric peak tetanic torque is reported as both raw peak torque and peak torque normalized to body mass (mN*m/g).

### Micro‐computed tomography (μCT) analysis

After euthanasia, the left femurs were stored in phosphate‐buffered saline (PBS) at −80°C. Following previously published methods,^(^ ^30^, ^31^, ^32^ ^)^ the midpoint of the femur diaphysis and the distal femur metaphysis were scanned in PBS at room temperature using ex vivo μCT scanner (Scanco μCT50, Scanco Medical AG, Bruttisellen, Switzerland) and then evaluated to assess cortical structure (eg, cortical thickness [Ct.Th], cross‐sectional bone area [Ct.Ar], cross‐sectional moment of inertia [Imin]), trabecular architecture (eg, bone volume fraction [BV/TV], trabecular thickness [Tb.Th], trabecular number [Tb.N], connectivity density [Conn.D]), and tissue mineral density of cortical and trabecular bone (Ct.TMD and Tb.TMD). For both scans (1.86 mm across the femur midpoint and 3.72 mm above the epiphysis), the scanner settings were as follows: an isotropic voxel size of 6 μm, peak X‐ray voltage of 70 kVp, tube current of 114 μA, integration time of 300 ms, sampling rate of 1160 acquisitions per 1000 projections per rotation of the tube holder. A 0.1‐mm‐thick aluminum filter was between the X‐ray beam and bone to narrow the energy spectrum and minimize beam‐hardening effects. Furthermore, a manufacturer‐recommended beam‐hardening correction (as part of the calibration to the hydroxyapatite phantom) was applied during each scan.

Post‐reconstruction of the scans by Scanco software, we applied a noise filter to the image stack (Gaussian smoothing parameters: standard deviation of the distribution, Sigma, and weighting of neighboring pixels, Support) of the diaphysis (Sigma = 0.8 and Support = 2) and metaphysis (Sigma = 0.2 and Support = 1). Then, segmentation of bone from soft tissue and air used different global density threshold for cortical bone (≥900.5 mgHA/cm^3^) and trabecular bone (≥429.4 mgHA/cm^3^) so that bone morphology and density parameters could be determined by Standard Scanco evaluation scripts.

### Three‐point bend testing

After the μCT evaluation of the femur mid‐diaphysis, each hydrated femur was loaded‐to‐failure at 3 mm/min in three‐point bending with a span of 8 mm using a mechanical testing system (DynaMight 8800, Instron, Norwood, MA, USA). During the mechanical test of each bone, the anterior side faced down and the medial side forward. The resulting force (Honeywell load cell, P/N 060‐0863‐02) versus displacement (linear variable differential transducer of the linear actuator) data were acquired at 50 Hz and processed using a custom Matlab (Mathworks, Natick, MA, USA) script to determine the stiffness, yield force, ultimate force, post‐yield displacement (PYD), and work‐to‐failure (area under the force versus displacement curve). The yield point was identified at the intersection of the force versus displacement curve and a linear curve with a slope of 0.9 × stiffness originating from the origin. Using equations from beam theory and μCT structural parameters, we estimated modulus and ultimate stress. Toughness was 3 × work‐to‐fracture/Ct.Ar/Span.^(^ ^33^ ^)^

### Immunohistochemistry (IHC)/fiber type and size analysis

The right gastrocnemii were excised, covered with O.C.T. Compound, and mounted at resting length. They were frozen in liquid nitrogen‐cooled isopentane and stored at −80°C until cryosectioning. Using a cryostat (HM525‐NX, Thermo Fisher Scientific, Waltham, MA, USA), 7‐μm‐thick sections were cut and air‐dried for 1 hour. Sections were stored at −20°C before IHC staining. Subsequently, for immunofluorescent assessment of muscle fiber type distribution and fiber type–specific cross‐sectional area (CSA), unfixed cryosections were incubated overnight at 4°C in primary antibodies against myosin heavy chain (MyHC) type 1 (dilution 1:100, Developmental Studies Hybridoma Bank [DHSB], Cat# BA‐D5 IgG2b), 2A (dilution 1:100, DSHB, Cat# SC‐71 IgG1), and 2B (dilution 1:100, DSHB, Cat# BF‐F3 IgM) in addition to laminin to visualize fiber borders (rabbit IgG, dilution 1:200; MilliporeSigma, Burlington, MA, USA, Cat# L9393). MyHC type 2X expression was inferred from unstained fibers. On the next day, slides were washed in PBS and incubated for 90 minutes at room temperature in fluorescent‐conjugated secondary antibodies (goat anti‐mouse IgG2b, Alexa Fluor 647 secondary antibody [1:250; Invitrogen, Carlsbad, CA, USA, Cat# A21242], goat anti‐mouse IgG1, Alexa Fluor 488 secondary antibody [1:500; Invitrogen, Cat# A21121], goat anti‐mouse IgM, Alexa Fluor 555 secondary antibody [1:250; Invitrogen, Cat# A21426], and goat anti‐rabbit IgG, AMCA‐conjugated secondary antibody [1:150; Vector Laboratories, Burlingame, CA, USA, Cat# Cl‐1000]) in PBS. Sections were post‐fixed in methanol before mounting. Images were captured at 10× with an upright microscope (AxioImager M1; Zeiss, Göttingen, Germany). MyoVision software was used for automated analysis of fiber‐type distribution, and fiber type–specific cross‐sectional area calculations.^(^ ^34^ ^)^

### Gastrocnemius RNA isolation and RT‐qPCR

For each mouse, the left gastrocnemius was excised, flash‐frozen in liquid nitrogen, and stored in −80°C until processing. RNA from the gastrocnemius was isolated after homogenizing the tissue in Tri‐Reagent (MilliporeSigma). After RNA isolation and cDNA synthesis (SuperScript IV Vilo Master Mix, Thermo Fisher Scientific, Waltham, MA, USA), relative gene expression was analyzed by quantitative PCR. Gene expression was quantified for Dickkopf Wnt Signaling Pathway Inhibitor 3 (*Dkk3*), Wnt family member 2 (*Wnt2*), Wnt family member 16 (*Wnt16*), Wnt family member 6 (*Wnt6*), mitochondrial pyruvate carrier 1 (*Mpc1*), nephronectin (*Npnt*), LDL receptor‐related protein 6 (*Lrp6*) and frizzled class receptor 4 (*Fzd4*) using TaqMan assays (Thermo Fisher Scientific; Supplemental Table S1). *β‐actin* was used as reference gene (Supplemental Table S1).

### MC3T3 growth and Smad2 phosphorylation

Murine pre‐osteoblast cells (MC3T3‐E1, RIKEN Cell Bank, Tsukuba, Japan) were plated in 6‐well plates (150,000 cells/well) on a monolayer and cultured with growth medium (alpha‐MEM (Gibco, Cat# A10490‐01) supplemented with 10% fetal bovine serum (Seradigm, premium grade FBS, VWR, Radnor, PA, USA) and 1X penicillin/streptomycin (Gibco [10,000 U/mL], Cat# 15140122]) every other day until confluent. Cells were then placed in low serum (1% FBS) for 6 hours. Subsequently, cells were stimulated with recombinant myostatin (250 ng/mL, R&D Systems, Cat# 788‐G8) with or without REGN647 myostatin antibody (1.5 μg/mL – 1:1 molar ratio to myostatin) for 45 minutes and subsequently lysed in cold RIPA buffer and processed for protein quantification with a BCA kit. Samples were run on SDS‐PAGE gels (Bio‐Rad, Hercules, CA, USA), which were transferred to PVDF membrane. After blocking in 5% nonfat dry milk in 1X tris‐buffered saline with Tween 20 (TBST), the membrane was incubated overnight at 4°C in anti‐pSMAD2 (1:1000, Cell Signaling [Danvers, MA, USA], Phospho‐SMAD2 [Ser465/467], Cat# 3108) or anti‐SMAD2,3 (1:1000, Cell Signaling, SMAD2/3 [D7G7] XP, Cat# 8685) diluted in 5% bovine serum albumin in 1X TBST. After washing with TBST, the membrane was incubated in goat anti‐rabbit IgG (H + L) secondary antibody (Thermo Fisher Scientific, Cat# 32460) for 1 hour at room temperature and then developed using Clarity Max ECL substrate (Bio‐Rad).

### MC3T3 growth and differentiation for RT‐qPCR analysis

MC3T3‐E1 cells were plated in 6‐well plates (150,000 cells/well) on a monolayer and grown as per above. When cells reached 90% confluency, they were stimulated with recombinant myostatin (250 or 500 ng/mL, R&D Systems, Cat# 788‐G8) or vehicle (4 mM HCl) for 24 hours. Cells were collected and processed for RNA isolation using RNA extraction kit (Quick‐RNA Miniprep Kit, Zymo Research, Irvine, CA, USA, Cat# R1051). Subsequently, cDNA synthesis was done using SuperScript IV Vilo Master Mix (Thermo Fisher Scientific). Genes of interest included Runt‐related transcription factor 2 (*Runx2*) and Sp7 transcription factor (*Sp7* or *Osx*), with *cyclophilin* used as reference gene. In separate experiments, MC3T3 cells were plated and grown as per above. When they reached ~90% confluency, they were maintained undifferentiated or with cocktail (10 mM β‐glycerophosphate [Sigma‐Aldrich], 50 μg/mL L‐ascorbic acid [Sigma‐Aldrich]) to induce differentiation and treated with either recombinant myostatin (250 ng/mL, R&D Systems, Cat# 788‐G8) or vehicle (4 mM HCl) for 2 weeks. Cells were harvested in Tri Reagent (Sigma‐Aldrich) and processed for RT‐qPCR as per above. Reference genes included Glyceraldehyde‐3‐phosphate dehydrogenase (*Gapdh*) and hypoxanthine‐guanine phosphoribosyl transferase (*Hprt*) and interest genes Alkaline phosphatase (*Alp*), Osteocalcin (*Bglap*), and Type 1 Collagen (*Cola1*) (Supplemental Table S1).

### Calcium staining

MC3T3 cells were plated in 12‐well plates and grown as per above. When they reached ~90% confluency, they were differentiated as per above for 2 weeks with or without recombinant myostatin (250 ng/mL). Calcium deposits were quantified with silver nitrate (Von Kossa staining).

### Statistical analysis

We summarize the mouse data using means and the standard deviation for continuous variables and frequencies and percentages for categorical variables. In understanding the differences among the six groups, we compared the average values and represented this using graphics. We used the one‐way analysis of variance (ANOVA) for comparing multiple groups and the analysis of covariance (ANCOVA) to understand the contribution of other covariates in the general linear model. The Tukey method allowed us to account for multiple comparisons across the six mouse groups and Bonferroni for multiple selected comparisons for gene expression data. For the in vivo experiments, one‐way ANOVA was used to determine differences among groups for each outcome variable. The general linear model technique compares the six groups while adjusting for the contribution of the risk factors associated with each outcome variable. We controlled for the effect of the risk factors like mouse body weight. We conduct all statistical hypothesis tests at the standard 5% significance level with a rejection of the null hypothesis for *p* values >5%. The SAS version 9.4 (TS1M1 SAS Institute Inc, Cary, NC, USA) statistical software and GraphPad (La Jolla, CA, USA) Prism version 9.5.1 are used for all analyses.

## Results

### Myostatin antibody (MyoAb) resulted in higher body weight, lean mass, and gastrocnemius weight in diabetic mice

**Fig. 1:**
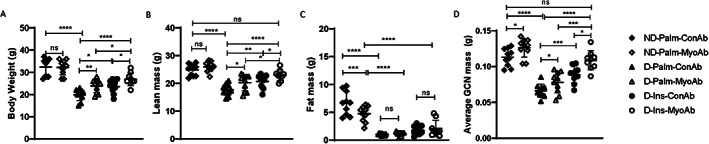
Effects of streptozotocin (STZ)‐induced diabetes, insulin, and MyoAb treatment on body weight (*A*), lean mass (*B*), and fat mass (*C*), as measured by Echo MRI, and average gastrocnemius (GCN) mass (*D*) at study end. Data presented as individual points with mean ± SD. *p < 0.05, **p < 0.01, ***p < 0.001, ****p < 0.0001, ns = not significant.

Baseline weight before induction of diabetes was not different among groups (data not shown). Diabetic mice had lower whole‐body, lean, and fat mass 1 week after STZ injections, before any treatment (data not shown). At study end, D mice had significantly lower body weight, lean mass, fat mass, and average gastrocnemius mass compared with ND mice (Fig. 1*A–D*, *p* < 0.0001). In D mice, myostatin inhibitory antibody (MyoAb) treatment for 8 weeks resulted in higher body weight (D‐Palm‐MyoAb versus D‐Palm‐ConAb, 23.9 g versus 19.9 g, *p* = 0.008, Fig. 1*A*), higher lean mass (D‐Palm‐MyoAb versus D‐Palm‐ConAb, 20.3 g versus 17.7 g, *p* = 0.014, Fig. 1*B*), and higher average gastrocnemius weight (D‐Palm‐MyoAb versus D‐Palm‐ConAb, 0.078 g versus 0.066 g, *p* = 0.045, Fig. 1*D*) when compared with control antibody treatment. Similarly, insulin treatment resulted in higher body weight (Fig. 1*A*), higher lean mass (Fig. 1*B*), and higher average gastrocnemius weight (Fig. 1*D*). The combination treatment of insulin and MyoAb (D‐Ins‐MyoAb) was superior in improving body weight, lean mass, and average gastrocnemius weight compared with MyoAb (D‐Palm‐MyoAb) or insulin (D‐Ins‐ConAb) alone (Fig. 1*A*, *B*, *D*). Moreover, lean mass and average gastrocnemius weight in D mice treated with the insulin/MyoAb combination were similar to ND mice (Fig. 1*B*, *D*). Fat mass was lower with diabetes (ND‐Palm‐ConAb versus D‐Palm‐ConAb, 6.66 g versus 1.07 g, *p* = <0.0001, Fig. 1*C*), but no differences were noted between all diabetic mice, irrespective of insulin or antibody treatment (Fig. 1*C*). ND mice treated with myostatin antibody had lower fat mass compared with ND mice on control antibody (Fig. 1*C*).

### MyoAb, contrary to insulin, did not alter glycemic control or bone formation markers P1NP and CTX‐I in diabetic mice

**Fig. 2:**
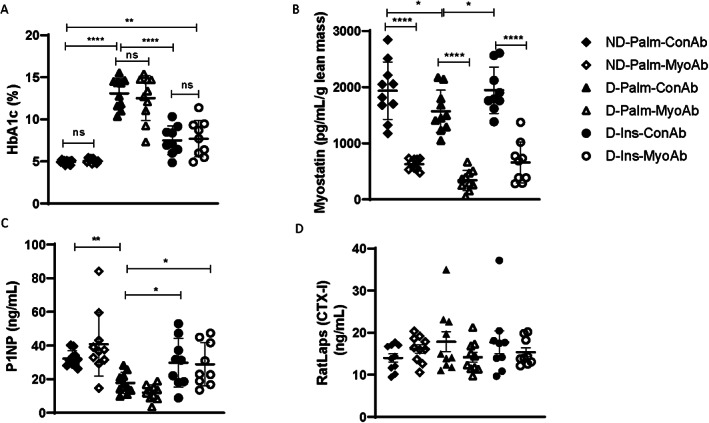
Effects of streptozotocin (STZ)‐induced diabetes, insulin, and MyoAb treatment on whole‐blood glycated hemoglobin (HbA1c) (*A*), serum myostatin normalized to body weight (*B*), serum Procollagen 1 Intact N‐Terminal Propeptide (P1NP) (*C*), and serum RatLaps (CTX‐I) (*D*) at 8 weeks. Data presented as individual points with mean ± SD. *p < 0.05, **p < 0.01, ***p < 0.001, ****p < 0.0001, ns = not significant.

D mice had higher glycated hemoglobin than ND mice, and insulin treatment was associated with significantly improved glycemic control, although it did not normalize glycated hemoglobin (D‐Ins‐ConAb versus ND‐Palm‐ConAb, 7.5% versus 5.0%, Fig. 2*A*). There was no effect of myostatin antibody treatment on glycemic control (HbA1c). Systemic myostatin adjusted for lean mass was lower in untreated diabetic mice (D‐Palm‐ConAb, 1572 pg/mL/g lean mass) compared with non‐diabetic mice (ND‐Palm‐ConAb, 1937 pg/mL/g lean mass, *p* = 0.026) or diabetic mice on insulin (D‐Ins‐ConAb, 1945 pg/mL/g lean mass, *p* = 0.027, Fig. 2*B*). Bone formation marker P1NP was significantly lower in D mice compared with ND mice (Fig. 2*C*). Insulin treatment resulted in higher P1NP (D‐Ins‐ConAb versus D‐Palm‐ConAb, 29.78 ng/mL versus 17.76 ng/mL, *p* = 0.028, Fig. 2*C*); however, myostatin antibody treatment did not change P1NP levels. Bone resorption marker RatLaps (CTX‐I) was not altered because of diabetes, insulin, or antibody treatment (Fig. 2*D*).

### MyoAb, insulin, or combination of insulin/MyoAb resulted in higher cross‐sectional area of type II muscle fibers in diabetic mice

**Fig. 3:**
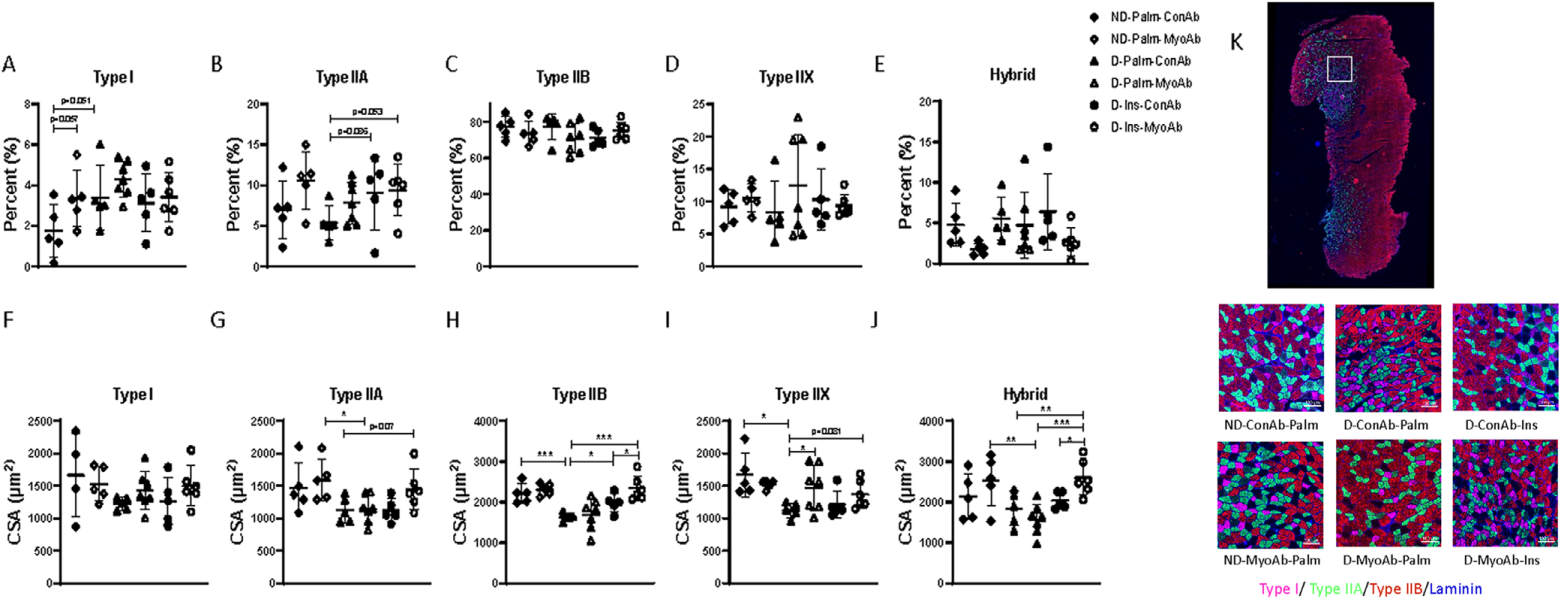
Effects of streptozotocin (STZ)‐induced diabetes, insulin, and MyoAb treatment on fiber type (*A*–*E*) and fiber cross‐sectional area (CSA) (*F*–*J*) in gastrocnemius muscle. Representative images from immunohistochemical analysis of gastrocnemius muscle cross sections for myosin heavy chain (MHC) type I (pink), type IIA (green), and type IIB (blue) (*K*). Unstained fibers are MHC type IIX. Scale bar = 100 μm. Data presented as individual points with mean ± SD. *p < 0.05, **p < 0.01, ***p < 0.001, ****p < 0.0001.

Skeletal muscle fiber analysis from gastrocnemius showed trends for higher Type I fiber percentage with diabetes (*p* = 0.051, Fig. 3*A*) and treatment with myostatin antibody trended toward higher Type I fiber percentage in ND mice (*p* = 0.057, Fig. 3*A*). There was also a trend for higher Type IIA fiber percentage with insulin treatment compared with control treatment (*p* = 0.053, Fig. 3*B*). No differences were found in Type IIB, Type IIX, or hybrid fiber percentage as a result of diabetes or any of the treatments (Fig. 3*C–E*). Diabetes had no effect on Type I fiber CSA (Fig. 3*F*); however, the average fiber CSA was lower as a result of diabetes for Type IIB and IIX fibers (Fig. 3*H*, *I*). Type IIA and hybrid fibers were lower with diabetes only in the MyoAb‐treated mice (Fig. 3*G*, *J*). In D mice, there was a trend for Type IIA fiber CSA improvement with combination of insulin/ MyoAb treatment (D‐Ins‐MyoAb versus D‐Palm‐ConAb, *p* = 0.07, Fig. 3*G*) reaching similar values to ND mice. Similarly, Type IIB fiber CSA was higher with insulin treatment alone (D‐Ins‐ConAb versus D‐Palm‐ConAb, 1979 μm^2^ versus 1633 μm^2^, *p* = 0.046, Fig. 3*H*) or combination of insulin/MyoAb (D‐Ins‐MyoAb versus D‐Palm‐ConAb, 2345 μm^2^ versus 1633 μm^2^, *p* = 0.0001, Fig. 3*H*), with combination treatment bringing the CSA to ND levels. Type IIX fiber CSA was higher with MyoAb treatment alone (D‐Palm‐MyoAb versus D‐Palm‐ConAb, 1472 μm^2^ versus 1123 μm^2^, *p* = 0.014, Fig. 3*I*) or combination of insulin/MyoAb (D‐Ins‐MyoAb versus D‐Palm‐ConAb, 1370 μm^2^ versus 1123 μm^2^, *p* = 0.081, Fig. 3*I*). Finally, hybrid fiber size was most responsive to the combination insulin/MyoAb treatment (D‐Ins‐MyoAb versus D‐Palm‐ConAb, 2603 μm^2^ versus 1841 μm^2^, *p* = 0.009, Fig. 3*J*).

### Wnt pathway genes were downregulated in skeletal muscle of mice with diabetes and MyoAb treatment resulted in downregulation of Wnt inhibitor *Dkk3*

**Fig. 4:**
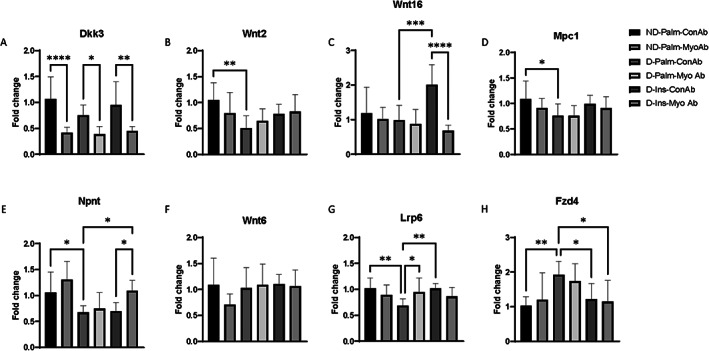
Effects of streptozotocin (STZ)‐induced diabetes, insulin, and MyoAb treatment on fold change (2^ddCT) of selected genes involved in the Wnt pathway in gastrocnemius muscle of mice. Relative expression normalized to ND‐Palm‐ConAb, reference gene *β‐Actin*. Data presented as mean ± SD. *p < 0.05, **p < 0.01, ***p < 0.001, ****p < 0.0001 (*p* values are Bonferroni corrected).

In the gastrocnemius muscle, several genes involved in the Wnt signaling pathway were downregulated by diabetes, including *Wnt2*, *Mpc1*, *Npnt*, and *Lrp6* (Fig. 4*B*, *D*, *E*, *G*), whereas *Fzd4* was higher in muscle from D mice (Fig. 4*H*). MyoAb treatment resulted in significant downregulation of *Dkk3* in all mice treated with myostatin antibody (Fig. 4*A*) and in upregulation of *Lrp6* (Fig. 4*G*) in D mice. Insulin upregulated *Wnt16* and *Lrp6* (Fig. 4*C*, *G*), whereas it downregulated *Fzd4* (Fig. 4*H*). Combination treatment (insulin/MyoAb) was associated with upregulation of *Npnt* (Fig. 4*E*) and downregulation of *Fzd4* (Fig. 4*H*).

### Muscle torque in diabetic mice was improved with combination treatment (insulin/MyoAb)

**Fig. 5:**
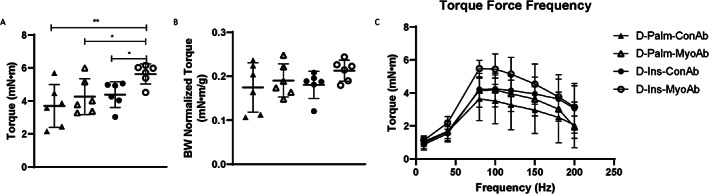
Effects of streptozotocin (STZ)‐induced diabetes, insulin, and MyoAb treatment on raw torque (*A*), torque normalized to body weight (*B*), and torque‐frequency curve (*C*) at study end. Data presented as individual points with mean ± SD (*A*, *B*) or mean ± SEM (*C*). *p < 0.05, **p < 0.01.

In D mice, results from in vivo contractile function analysis showed that combination treatment (insulin/MyoAb) resulted in significant improvement in muscle torque in D mice compared with control treatment (D‐Ins‐MyoAb versus D‐Palm‐ConAb, 5.63 mN*m versus 3.70 mN*m, *p* = 0.0016), whereas insulin or MyoAb treatment alone was not significantly different from control treatment (Fig 5*A*, *C*). However, this effect was not sustained when torque was normalized to body weight (Fig. 5*B*), indicating that this improvement in muscle strength is likely a result of increased gains in lean mass and not due to improved muscle quality.

### In diabetic mice, MyoAb treatment was beneficial for cortical bone structure and strength when used alone, whereas it was beneficial for trabecular bone when combined with insulin

**Fig. 6:**
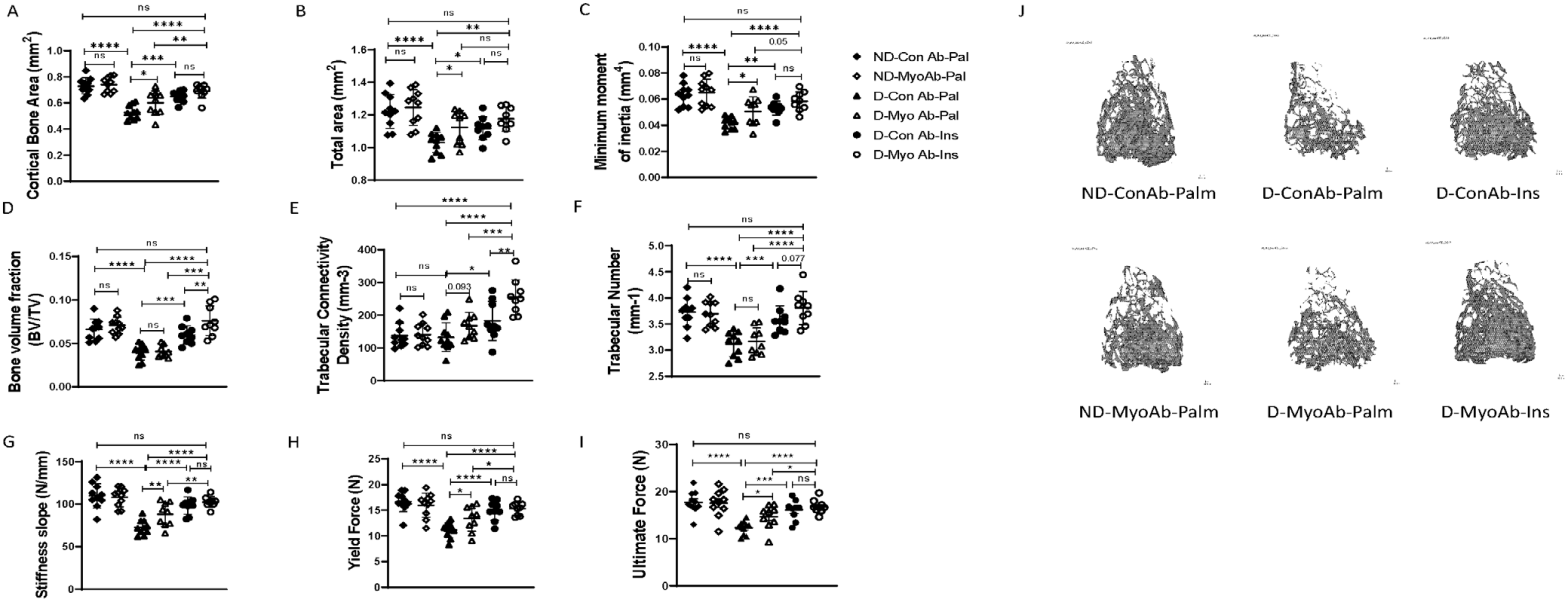
Effects of streptozotocin (STZ)‐induced diabetes, insulin, and MyoAb treatment on bone microarchitecture and whole‐bone biomechanics. Selected cortical properties, including cortical bone area (*A*), total area (*B*), minimum moment of inertia (*C*); selected trabecular properties including bone volume fraction (*D*), connectivity density (*E*), and trabecular number (*F*) as measured by μCT analysis. Selected biomechanical properties including stiffness (*G*), force at yielding (*H*), and ultimate force (*I*) as measured by three‐point bending test. Representative images of trabecular bone microarchitecture by μCT (*J*). Data presented as individual values with mean ± SD. *p < 0.05, **p < 0.01, ***p < 0.001, ****p < 0.0001, ns = not significant.

When evaluating bone microarchitecture, diabetes was associated with impaired bone parameters in cortical and trabecular bone (Fig. 6 and Table 1). In D mice, several cortical properties, such as cortical bone area, total area, and minimum moment of inertia of the mid‐diaphysis, were improved with MyoAb treatment (D‐Palm‐MyoAb) or insulin treatment alone (D‐Ins‐ConAb) compared with control treatment (D‐Palm‐ConAb) (Fig. 6 *A–C* and Table 1). Although combination treatment restored cortical bone area to similar levels as ND mice (D‐Ins‐MyoAb versus ND‐Palm‐ConAb, Fig. 6*A*), for some parameters, such as total area and minimum moment of inertia, combination treatment was not superior to MyoAb treatment (D‐Palm‐MyoAb) or insulin treatment alone (D‐Ins‐ConAb) (Fig. 6*B*, *C*). In D mice, contrary to insulin treatment, MyoAb treatment alone was not associated with improvements in trabecular properties, such as BV/TV, Conn.D, or Tb.N (Fig. 6*D–F*, *J* and Table 1). However, combination treatment of insulin with MyoAb (D‐Ins‐MyoAb) was associated with significant improvements in bone volume fraction and trabecular connectivity compared to insulin treatment alone (D‐Ins‐ConAb) (Fig. 6*D*, *E*, *J* and Table 1).

**Table 1:** Micro‐CT Trabecular and Cortical Parameters of Femur, Including Whole Bone Mechanical Properties of Femur From 3‐Point Bending Test

	ND‐palm‐ConAb	ND‐palm‐MyoAb	D‐palm‐ConAb	D‐palm‐MyoAb	D‐ins‐ConAb	D‐ins‐MyoAb	Diabetes effect (1 vs. 3)	Insulin effect (3 vs. 5)	MyoAb effect (3 vs. 4)	Insulin/MyoAb effect (3 vs. 6)	MyoAb additive effect (5 vs. 6)
Diaphysis/cortical bone											
Ma.V (mm^3^)	0.904 ± 0.09	0.927 ± 0.10	0.921 ± 0.09	0.960 ± 0.05	0.863 ± 0.08	0.889 ± 0.07	ns A	ns A	ns NS	ns A	ns NS
Imin (mm^4^)	0.063 ± 0.008	0.065 ± 0.010	0.042 ± 0.005	0.051 ± 0.011	0.053 ± 0.005	0.058 ± 0.007	d NS	b NS	a NS	d NS	ns NS
Ct. AR (mm^2^)	0.73 ± 0.06	0.74 ± 0.06	0.53 ± 0.05	0.60 ± 0.09	0.65 ± 0.04	0.69 ± 0.05	d NS	c B	a NS	d B	ns NS
Tt. AR (mm^2^)	1.22 ± 0.10	1.25 ± 0.11	1.03 ± 0.06	1.12 ± 0.10	1.12 ± 0.07	1.18 ± 0.07	d NS	a NS	a NS	b NS	ns NS
Ct. TMD (mgHA/cm^3^)	1284 ± 22	1291 ± 19	1294 ± 26	1285 ± 19	1287 ± 25	1267 ± 26	ns NS	ns NS	ns NS	a NS	ns NS
Ct. Po (%)	1.25 ± 0.23	1.28 ± 0.20	1.59 ± 0.16	1.54 ± 0.26	1.27 ± 0.18	1.33 ± 0.30	b A	b B	ns NS	a A	ns NS
Length (mm)	14.1 ± 0.2	14.2 ± 0.2	13.3 ± 0.3	13.6 ± 0.3	13.6 ± 0.3	13.8 ± 0.2	d A	a NS	a NS	d NS	ns NS
Bending strength (MPa)	279 ± 25	271 ± 32	275 ± 29	275 ± 23	290 ± 24	283 ± 26	ns NS	ns NS	ns NS	ns NS	ns NS
Modulus (GPa)	18.6 ± 1.2	17.9 ± 2.1	19.5 ± 3.7	18.8 ± 1.7	19.8 ± 1.2	18.9 ± 1.5	ns NS	ns NS	ns NS	ns NS	ns NS
Stiffness (N/mm)	110 ± 14	108 ± 11	73 ± 9	88 ± 14	98 ± 10	103 ± 6	d A	d C	b NS	d B	ns NS
Yield force (N)	16.7 ± 2.0	16.0 ± 2.4	11.1 ± 1.5	13.4 ± 2.5	15.0 ± 1.9	15.3 ± 1.2	d NS	d B	a NS	d A	ns NS
Ultimate force (N)	17.74 ± 2.4	17.54 ± 2.9	12.28 ± 1.5	14.69 ± 2.5	16.07 ± 2.2	16.82 ± 1.4	d NS	c A	a NS	d A	ns NS
Peak moment (N.mm)	35.5 ± 4.8	35.1 ± 5.8	24.9 ± 3.1	29.4 ± 5.1	32.1 ± 4.3	33.7 ± 2.9	d NS	c A	a NS	d NS	ns NS
Normalized PYD (mm)	0.031 ± 0.02	0.038 ± 0.02	0.025 ± 0.01	0.024 ± 0.01	0.029 ± 0.01	0.027 ± 0.01	ns NS	ns NS	ns NS	ns NS	ns NS
Metaphysis/trabecular bone											
Tb.BV/TV (mm^3^)	6.6 ± 1.2	7.1 ± 1.0	4.0 ± 0.9	4.1 ± 0.7	6.0 ± 1.0	7.6 ± 1.8	d NS	c B	ns NS	d D	b A
Conn.D (mm^−3^)	137.1 ± 37.9	141.0 ± 33.8	132.5 ± 44.2	168.3 ± 40.4	182.5 ± 59.6	255.6 ± 54.1	ns NS	a A	ns NS	d D	b B
SMI	2.72 ± 0.14	2.65 ± 0.15	2.70 ± 0.26	2.61 ± 0.10	2.59 ± 0.18	2.30 ± 0.15	ns NS	ns NS	ns NS	d C	c B
Tb.N (mm^−1^)	3.73 ± 0.27	3.70 ± 0.23	3.12 ± 0.24	3.17 ± 0.26	3.58 ± 0.27	3.81 ± 0.32	d B	c C	ns NS	d D	ns NS
Tb.Th (mm)	0.041 ± 0.002	0.043 ± 0.002	0.031 ± 0.007	0.030 ± 0.002	0.036 ± 0.003	0.037 ± 0.003	d NS	b NS	ns A	b NS	ns NS
Tb.Sp (mm)	0.277 ± 0.188	0.280 ± 0.017	0.327 ± 0.025	0.322 ± 0.024	0.287 ± 0.020	0.273 ± 0.021	d B	C B	ns NS	d D	ns NS
Tb.TMD (mgHA/cm^3^)	890.1 ± 17.5	889.9 ± 15.3	841.1 ± 41.3	823.3 ± 14.8	867.9 ± 24.2	855.8 ± 25.7	d	d	ns	d	ns

Data is presented as mean ± SD.

BV/TV = bone volume/tissue volume; Conn.D = connectivity density; Ct.Ar = cortical area; Ct.Po = cortical porosity; Ct.TMD = cortical tissue mineral density; Imin = minimal moment of inertia; Ma.V = marrow volume; PYD = post‐yield deflection; SMI = structural model index; Tb.N = trabecular number; Tb.Sp = trabecular separation; Tb.Th = trabecular thickness; Tb.TMD = trabecular tissue mineral density; Tt.Ar = total cross‐sectional area; Yield force = the value of load at the yield point; Ultimate force = maximum value of load attained during the test.

a = *p* < 0.05, b = *p* < 0.01, c = *p* < 0.001, d = *p* < 0.0001, ns = non‐significant. After adjusting for body weight: A = *p* < 0.05, B = *p* < 0.01, C = *p* < 0.001, D = *p* < 0.0001, NS = not significant.

Finally, three‐point bending tests showed significantly decreased stiffness and yield force of femurs of diabetic mice compared with non‐diabetic mice (Fig 6*G*, *H* and Table 1). Treatment with the MyoAb compared with control treatment resulted in greater stiffness (D‐Palm‐MyoAb versus D‐Palm‐ConAb, *p* = 0.004, Fig. 6 *G* and Table 1), greater yield force (D‐Palm‐MyoAb versus D‐Palm‐ConAb, *p* = 0.015, Fig. 6 *H* and Table 1), and greater ultimate force (D‐Palm‐MyoAb versus D‐Palm‐ConAb, *p* = 0.022, Fig. 6 *I* and Table 1) in the femur. Similar, but greater, changes were found with insulin treatment or combination treatment (insulin/MyoAb) (Fig. 6 *H* and Table 1). The bending strength (ie, ultimate stress) and modulus of the femurs were not different with diabetes or any of the treatments (Table 1). When adjusting for mouse body weight, several of the changes in bone microarchitecture and biomechanical properties observed with diabetes or any of the treatments were no longer statistically significant, indicating that many of the differences are dependent on mouse body weight (Table 1).

### Myostatin directly stimulated MC3T3‐E1 pre‐osteoblasts and negatively affected their differentiation and mineralization potential

**Fig. 7:**
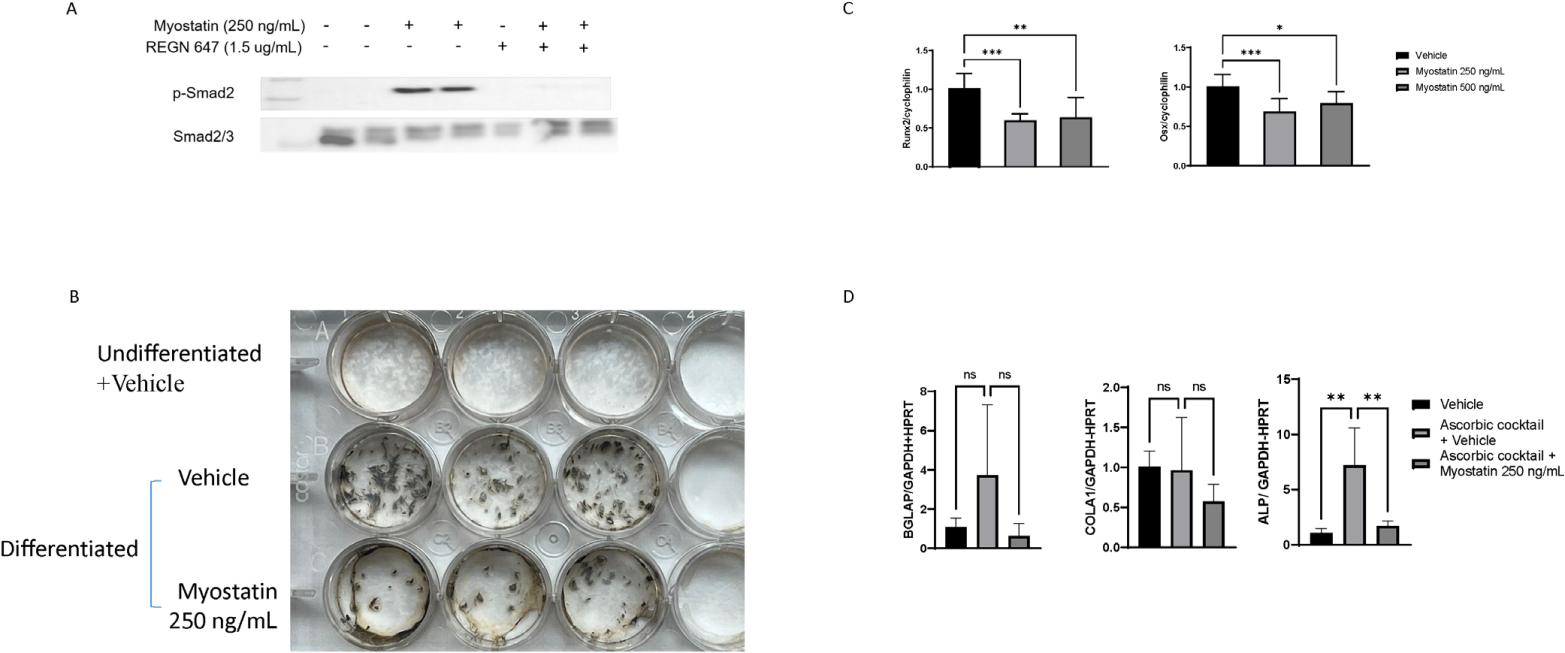
Effects of myostatin on pre‐osteoblastic (MC3T3‐E1) cells. Western blot showing that myostatin‐induced Smad 2 phosphorylation in MC3T3‐E1 cells is inhibited by the myostatin‐blocking antibody REGN647 (*A*). Myostatin inhibits mineralization of MC3T3‐E1 cells during differentiation for 2 weeks (*B*). Myostatin downregulates genes involved in osteogenesis of MC3T3‐E1 cells (*C*) and genes involved in osteoblast function during MC3T3‐E1 differentiation (*D*). (*C*) Results are average of 3 separate experiments, relative expression normalized to vehicle, reference gene: *cyclophilin*. (*D*) Results are average of 2 separate experiments, relative expression normalized to vehicle, reference genes: *Gapdh* and *Hprt*. Data are presented as mean ± SD. *p < 0.05, **p < 0.01, ***p < 0.001 (*p* values are Bonferroni corrected).

Our in vitro results showed that recombinant myostatin stimulated MC3T3‐E1 pre‐osteoblasts and induced phosphorylation of Smad2, which was inhibited by the myostatin antibody used in our in vivo studies (REGN647) (Fig. 7*A*). Additionally, myostatin suppressed mineralization evidenced by a decrease in calcium deposits (Von Kossa stain) in MC3T3‐E1 cells exposed to myostatin for 2 weeks during differentiation (Fig. 7*B*). Furthermore, genes involved in osteoblast differentiation and function such as *Runx2*, *Osx*, and *Alp* were downregulated by myostatin, indicating a direct effect on osteoblasts (Fig. 7*C*, *D*).

## Discussion

With this study, we sought to evaluate whether pharmacologic inhibition of myostatin with a myostatin inhibitory antibody (REGN647) improves muscle and bone mass, as well as muscle and bone strength, in a mouse model of insulin‐deficient diabetes. Previous studies targeting myostatin have shown promise as they have improved bone and muscle regenerative signaling pathways in mice with insulin‐deficient diabetes. However, one of these studies used exercise as the method for myostatin downregulation,^(^ ^26^ ^)^ which is not specific for myostatin inhibition; another study used follistatin or Alk5 inhibitor,^(^ ^25^ ^)^ which inhibit TGF‐β signaling and therefore are not as specific as the myostatin inhibitory antibody used in this study. Additionally, these studies did not evaluate muscle strength, bone strength, or bone microarchitecture and did not assess the effects of myostatin inhibition when combined with insulin treatment.

This is the first comprehensive study to report on the effects of myostatin pharmacologic inhibition on an insulin‐deficient model of diabetes and describe these effects on both muscle and bone. Our results show that myostatin inhibition with REGN647 significantly improved body mass, lean mass, cortical bone properties, and bone biomechanical properties in diabetic mice. However, myostatin inhibition was not sufficient to produce improvements in muscle strength. Additionally, it did not have any effect on glycemia or serum bone turnover markers P1NP or CTX‐I. The effects of REGN647 on the musculoskeletal phenotype of the diabetic mice were observed despite them having lower systemic myostatin levels compared with non‐diabetic mice. Insulin treatment resulted in higher body mass, lean mass, and bone formation marker P1NP as well as improved bone microarchitecture and biomechanical properties but, similar to REGN647, was not sufficient to improve muscle strength. Similarly, the insulin treatment protocol used in this study was not sufficient to completely normalize blood glucose concentration as evidenced by the higher HbA1c in diabetic mice on insulin therapy compared with non‐diabetic mice. This observation suggests that deficits in glucose homeostasis and muscle strength in diabetic mice might require more intensive insulin therapy to restore these parameters to normal levels. More importantly, the combination of insulin and myostatin antibody treatment in our study was associated with greater effects compared with either treatment alone in improving muscle mass (secondary to restoring muscle atrophy of type IIB and hybrid fibers), muscle strength, and trabecular bone properties. Individuals with T1D are on insulin therapy; however, they continue to have deficits in muscle and bone strength despite insulin treatment. This study supports the use of a pharmacologic myostatin inhibitor in addition to insulin as the combination treatment appears to remedy these deficits.

Inhibition of myostatin in our diabetic mice improved muscle and bone parameters without affecting glycemic control. The effects of myostatin on glycemic control and its interaction with insulin, systemically but also in different tissues (eg, bone), is not well understood. Other studies have reported improvements in insulin sensitivity and glycemia with myostatin inhibition primarily in obese and type 2 diabetes animal models^(^ ^35^, ^36^, ^37^ ^)^ but also in type 1 diabetes animal models.^(^ ^38^ ^)^ However, these animal studies utilized different models of diabetes and/or myostatin inhibition than the present study and that could explain the discrepancy in the findings relating to glycemic control. In this study, cortical bone properties in diabetic mice were not further improved with combination treatment, whereas femur biomechanical properties were further improved only when adding insulin to REGN647. This suggests that insulin and REGN647 might be targeting different signaling pathways that are altered with diabetes in skeletal muscle and trabecular bone, but similar pathways in cortical bone as their effect was not additive in this bone compartment. Further studies focusing on myostatin signaling in bone cells and its interaction with insulin are necessary to understand its direct effects on diabetic bone.

Our findings are in alignment with previous studies in animals with insulin‐deficient diabetes that have shown deficits in muscle mass^(^ ^39^, ^40^ ^)^ and bone mass,^(^ ^41^, ^42^, ^43^ ^)^ as well as impaired skeletal muscle function^(^ ^40^, ^44^ ^)^ and bone strength.^(^ ^45^, ^46^, ^47^ ^)^ Furthermore, we have previously shown that insulin improves bone formation markers, cortical and trabecular bone, and its biomechanical properties in insulin‐deficient diabetic animals,^(^ ^42^ ^)^ which was confirmed in this study. Although our study did not identify changes in bone turnover markers with myostatin inhibition at the end of the study, gains in cortical bone and trabecular bone by μCT were observed with the myostatin antibody alone or in combination with insulin. This discrepancy could be explained by the bone turnover markers reflecting bone cell activity only at a single time point at the conclusion of the study, whereas bone morphology by μCT can assess effects on the bone over time. Additionally, the cortical compartment where the myostatin antibody had the strongest effect is not as rich in bone cells as trabecular bone, therefore changes in cortical bone might not be reflected in changes in serum bone turnover markers. Other studies have reported on the use of REGN647 or similar antibodies in animal models of osteogenesis imperfecta^(^ ^28^, ^48^ ^)^ or aging^(^ ^27^ ^)^ and have assessed its effects on muscle and bone. These studies showed gains in muscle mass and muscle force production^(^ ^27^ ^)^ and improvements in bone phenotype.^(^ ^28^, ^48^ ^)^ Another effect of myostatin inhibition that has previously been shown is lower fat mass.^(^ ^49^ ^)^ In the present study, REGN647 reduced fat mass in the non‐diabetic mice but not in diabetic mice. Nevertheless, this effect might be beneficial in humans with T1D as a significant portion of adults with T1D (~38%) in the United States are also obese.^(^ ^50^ ^)^

Wnt has been implicated in myogenesis and muscle homeostasis.^(^ ^51^ ^)^ Specifically, the Wnt signaling pathway is important during embryonic development, as it controls myogenic regulatory factors (MRFs) and in adult skeletal muscle, where it regulates the differentiation of muscle stem cells and the growth of muscle fibers.^(^ ^51^ ^)^ STZ‐induced diabetes has been associated with downregulation of Wnt activity in mouse plantaris muscle.^(^ ^52^ ^)^ Furthermore, Dickkopf 3 (*Dkk3*), which is a negative regulator of Wnt signaling, has been implicated in muscle atrophy^(^ ^53^ ^)^ and is suppressed by myostatin inhibition.^(^ ^27^ ^)^ In this study, we focused on how insulin‐deficient diabetes and or myostatin inhibition affected genes involved in the Wnt pathway in skeletal muscle. We found that Wnt pathway–related genes are downregulated with insulin‐deficient diabetes (Fig. 4), whereas systemic myostatin inhibition with REGN647 resulted in lower *Dkk3* gene expression. In addition, Wnt co‐receptor *Lrp6* and *Npnt* gene expression was altered by treatment with REGN647 in a diabetic environment (Fig. 4). Interestingly, the expression of some of these genes is also altered by insulin or the combination of insulin/REGN647. Taken together, these results support the hypothesis that myostatin inhibition could prevent muscle atrophy in a condition of insulin‐deficient diabetes.

Our in vitro studies showed direct myostatin action in osteoblasts (MC3T3‐E1) and evidence of activation of Smad 2/3. Smad 2/3 is an important component of the classical myostatin signaling pathway in muscle^(^ ^54^ ^)^ and Smad 2 phosphorylation by myostatin in MC3T3 confirms that myostatin can activate the same classical pathway in osteoblasts. Additionally, we observed that myostatin inhibits bone mineralization and downregulates genes involved in osteogenesis and bone maturation. These findings are in agreement with previous studies that have shown decreased mineralization potential and decrease in osteogenic genes of bone marrow stromal cells^(^ ^55^ ^)^ or primary osteoblasts^(^ ^56^ ^)^ in the presence of myostatin. Lastly, we show that REGN647 can inhibit myostatin‐induced phosphorylation of Smad2 in MC3T3‐E1 cells.

Our study has several limitations. Only male mice were included in our study, therefore we cannot comment on sex differences at this time. Additionally, we cannot comment on whether the myostatin inhibitory antibody was also inhibiting to Growth and Differentiation factor 11 (GDF‐11); however, other studies reporting on this particular antibody have shown very weak binding to GDF‐11 compared with myostatin.^(^ ^28^ ^)^ Furthermore, we have not reported on affected signaling pathways in bone tissue from our mice, therefore we cannot identify a direct mechanism for myostatin inhibition on bone in this study; perhaps the changes in skeletal muscle Wnt signaling or other signaling pathways are sufficient to alter the paracrine microenvironment in bone, resulting in the observed bone phenotype in our mice. Finally, we have not conducted in vitro experiments on osteoclasts or osteocytes, which could potentially also be affected by pharmacologic inhibition of myostatin. These will need to be addressed in future studies.

With this study, we show that pharmacologic inhibition of myostatin with REGN647 improves body weight, lean mass, and muscle strength in insulin‐deficient mice, despite suboptimal glycemic control. Additionally, it improves cortical bone properties and bone strength with less of an effect on trabecular bone, unless combined with insulin. Further studies on the interaction of myostatin with insulin signaling in bone and muscle tissue are needed to determine whether the effects of myostatin inhibition are acting directly or indirectly on these tissues. Pharmacologic myostatin inhibition could be a potential future target to improve deficits in muscle and bone in those with type 1 diabetes.

## Author Contributions

**Robert Clay Bunn:** Conceptualization; data curation; formal analysis; investigation; methodology; project administration; supervision; visualization; writing – original draft; writing – review and editing. **Reuben Adatorwovor:** Data curation; formal analysis; methodology; writing – review and editing. **Rebecca R. Smith:** Data curation; investigation; writing – review and editing. **Philip Ray:** Data curation; formal analysis; investigation; methodology; writing – review and editing. **Sarah E. Fields:** Data curation; formal analysis; investigation; writing – review and editing. **Alexander R. Keeble:** Data curation; investigation; writing – review and editing. **Christopher S. Fry:** Data curation; formal analysis; methodology; resources; visualization; writing – review and editing. **Sasidhar Uppuganti:** Data curation; investigation; methodology; visualization; writing – review and editing. **Jeffry S. Nyman:** Conceptualization; data curation; formal analysis; methodology; supervision; validation; writing – review and editing. **John Fowlkes:** Conceptualization; formal analysis; methodology; resources; supervision; writing – review and editing. **Evangelia Kalaitzoglou:** Conceptualization; data curation; formal analysis; funding acquisition; investigation; methodology; project administration; resources; supervision; visualization; writing – original draft; writing – review and editing.

## Disclosures

The authors declare no conflicts of interest.

### Peer Review

The peer review history for this article is available at https://www.webofscience.com/api/gateway/wos/peer-review/10.1002/jbm4.10833.

## Supporting information


**Supplemental Table S1.** Primer sequences or TaqMan Assay for Rt‐qPCR genes, and their role in musculoskeletal tissues.

## Data Availability

All data that support the findings of this study are presented in the main article or in the supplemental section.
