# Supplementary material for: Pharmacologic Inhibition of Myostatin With a Myostatin Antibody Improves the Skeletal Muscle and Bone Phenotype of Male Insulin‐Deficient Diabetic Mice
Source: JBMR Plus. 2023 Oct 26;7(11):e10833. doi: 10.1002/jbm4.10833 (PMC10652179; doi:10.1002/jbm4.10833)
Supplement: Supplementary file 1 — Supplemental Table S1. Primer sequences or TaqMan Assay for Rt‐qPCR genes, and their role in musculoskeletal tissues. [file JBM4-7-e10833-s001.pdf]

Supplemental Table 1

Primer sequences or TaqMan Assay for Rt-qPCR genes, and their role in musculoskeletal tissues

Gene	Forward	Reverse	ThermoFisher TaqMan assay ID	Role
Runx2	CGCACGACAACCG CACCAT	CAGCACGGAGCAC AGGAAGTT	-	Osteogenesis (1)
Osx	CTCGTCTGACTGCC TGCCTAG	GCGTGGATGCCTGC CTTGTA	-	Osteogenesis (1)
Cyclophilin	GCATACGGGTCCT GGCATCTTGTCC	ATGGTGATCTTCTTG CTGGTCTTGC	-	Reference gene
Alp	-	-	Mm00475834_m1	Osteoblast differentiation and mineralization (1)
Bglap	-	-	Mm03413826_m1	Osteoblast differentiation (1)
Col1a1	-	-	Mm00801666_m1	Osteoblast differentiation (1)
Hprt			Mm00446968_m1	Reference gene
Gapdh			Mm99999915_g1	Reference gene
Dkk3	-	-	Mm00443800_m1	Antagonist of Wnt pathway, increases β-catenin, induces muscle atrophy (2)
Wnt2	-	-	Mm00470018_m1	Agonist of Wnt pathway, induces fibrosis in skeletal muscle (3)
Wnt6	-	-	Mm00437353_m1	Agonist of Wnt Signaling, involved in myoblast cell fusion/differentiation (4)
Wnt16			Mm00446420_m1	Agonist of Wnt signaling, involved in bone and muscle morphology (5)
Mpc1			Mm01316203_g1	Glucose oxidation, potential inhibitor of Wnt pathway (6)
Npnt			Mm00473794_m1	Target of Wnt pathway, extracellular matrix protein, promotes myoblast fusion (7, 8)

For Review Only

Lrp6			Mm00999795_m1	Wnt Co-receptor, affects distribution and fiber type size (9)
Fzd4			Mm00433382_m1	Wnt receptor, affects myogenesis and skeletal muscle regeneration (10)
β-actin (reference gene)	-	-	Mm01205647_g1	Reference gene

1. <kirkham_chapter_01.pdf>.
2. Yin J, Yang L, Xie Y, Liu Y, Li S, Yang W, et al. Dkk3 dependent transcriptional regulation controls age related skeletal muscle atrophy. Nat Commun. 2018;9(1):1752.
3. Hidestrand M, Richards-Malcolm S, Gurley CM, Nolen G, Grimes B, Waterstrat A, et al. Sca-1-expressing nonmyogenic cells contribute to fibrosis in aged skeletal muscle. J Gerontol A Biol Sci Med Sci. 2008;63(6):566-79.
4. Hitchins L, Fletcher F, Allen S, Dhoot GK. Role of Sulf1A in Wnt1- and Wnt6-induced growth regulation and myoblast hyper-elongation. FEBS Open Bio. 2013;3:30-4.
5. Watson CJ, Tang WJ, Rojas MF, Fiedler IAK, Morfin Montes de Oca E, Cronrath AR, et al. wnt16 regulates spine and muscle morphogenesis through parallel signals from notochord and dermomyotome. PLoS Genet. 2022;18(11):e1010496.
6. Tian GA, Xu CJ, Zhou KX, Zhang ZG, Gu JR, Zhang XL, et al. MPC1 Deficiency Promotes CRC Liver Metastasis via Facilitating Nuclear Translocation of beta-Catenin. J Immunol Res. 2020;2020:8340329.
7. Lamarche E, AlSudais H, Rajgara R, Fu D, Omaiche S, Wiper-Bergeron N. SMAD2 promotes myogenin expression and terminal myogenic differentiation. Development. 2021;148(3).
8. Sun Y, Kuek V, Qiu H, Tickner J, Chen L, Wang H, et al. The emerging role of NPNT in tissue injury repair and bone homeostasis. J Cell Physiol. 2018;233(3):1887-94.
9. Gessler L, Kurtek C, Merholz M, Jian Y, Hashemolhosseini S. In Adult Skeletal Muscles, the Co-Receptors of Canonical Wnt Signaling, Lrp5 and Lrp6, Determine the Distribution and Size of Fiber Types, and Structure and Function of Neuromuscular Junctions. Cells. 2022;11(24).
10. Zhang D, Yin L, Lin Z, Yu C, Li J, Ren P, et al. miR-136-5p/FZD4 axis is critical for Wnt signaling-mediated myogenesis and skeletal muscle regeneration. J Cell Physiol. 2023.

For Review Only
